# Biallelic Variants in *CFAP61* Cause Multiple Morphological Abnormalities of the Flagella and Male Infertility

**DOI:** 10.3389/fcell.2021.803818

**Published:** 2022-01-31

**Authors:** Ao Ma, Aurang Zeb, Imtiaz Ali, Daren Zhao, Asad Khan, Beibei Zhang, Jianteng Zhou, Ranjha Khan, Huan Zhang, Yuanwei Zhang, Ihsan Khan, Wasim Shah, Haider Ali, Abdul Rafay Javed, Hui Ma, Qinghua Shi

**Affiliations:** Division of Reproduction and Genetics, First Affiliated Hospital of USTC, Hefei National Laboratory for Physical Sciences at Microscale, School of Basic Medical Sciences, Division of Life Sciences and Medicine, Biomedical Sciences and Health Laboratory of Anhui Province, CAS Center for Excellence in Molecular Cell Science, Collaborative Innovation Center of Genetics and Development, University of Science and Technology of China, Hefei, China

**Keywords:** male infertility, multiple morphological abnormalities of flagella, central pair, calmodulin- and spoke-associated complex, Cfap61

## Abstract

Multiple morphological abnormalities of the flagella (MMAF) can lead to male infertility due to impaired sperm motility and morphology. Calmodulin- and spoke-associated complex (CSC) are known for their roles in radial spoke (RS) assembly and ciliary motility in Chlamydomonas, while the role of cilia- and flagella-associated protein 61 (CFAP61), a mammalian ortholog of the CSC subunits, in humans is yet unknown. Here, we recruited three unrelated Pakistani families comprising of 11 infertile male patients diagnosed with MMAF. *CFAP61* variants, c.451_452del (p.I151Nfs*4) in family 1 and c.847C > T (p.R283*) in family 2 and 3, were identified recessively co-segregating with the MMAF phenotype. Transmission electron microscopy analyses revealed severe disorganized axonemal ultrastructures, and missings of central pair, RSs, and inner dynein arms were also observed and confirmed by immunofluorescence staining in spermatozoa from patients. CFAP61 and CFAP251 signals were absent from sperm tails of the patients, which suggested the loss of functional CSC in sperm flagella. Altogether, our findings report that homozygous variants in *CFAP61* are associated with MMAF and male infertility, demonstrating the essential role of this gene in normal sperm flagellum structure in humans.

## Introduction

Infertility is a worldwide health concern, approximately affecting 15% of couples (Jiao et al., 2021). Male factors account for about 50% of infertility cases (Jiao et al., 2021). Multiple morphological abnormalities of the flagella (MMAF) is characterized as a severe form of asthenozoospermia with absent, short, bent, coiled flagella, and flagella of irregular caliber (Ben Khelifa et al., 2014). MMAF is mainly caused by defects in the axoneme in sperm flagella, such as missing of central pair (CP), disorganization of microtubule doublets (MTDs), and/or outer dense fibers (ODFs). So far, whole-exome sequencing (WES) studies have identified at least 33 genes with pathogenic mutations responsible for MMAF, such as *CFAP43*, *CFAP44*, *CFAP47*, *CFAP65*, *CFAP69*, *CFAP70*, *CFAP91*, *CFAP251*, *DNAH17* etc (He et al., 2019; Liu et al., 2020; Lv et al., 2020; Martinez et al., 2020; Ni et al., 2020; Sha et al., 2020; Zhang B. et al., 2021; Zhang J. et al., 2021; Cong et al., 2021; Guo et al., 2021; Liu et al., 2021; Lorès et al., 2021; Ma et al., 2021; Shen et al., 2021; Touré et al., 2021; Tu et al., 2021). However, only approximately 60% of MMAF cases can be explained by the current genetic findings (Liu et al., 2021), demonstrating the necessity for further studies to explore new genetic factors involved in MMAF.

Calmodulin- and spoke-associated complex (CSC) is known for its essential role in radial spoke (RS) assembly and ciliary/flagellar motility (Dymek and Smith, 2007; Dymek et al., 2011; Heuser et al., 2012; Urbanska et al., 2015). CSC proteins were firstly identified in *Chlamydomonas reinhardtii*, when immunoprecipitation-mass spectrometry was used to find proteins interacting with Calmodulin (CaM). Disruption of CSC proteins in *C. reinhardtii* could lead to impaired flagellar motility and axoneme structure defects, including partially loss of radial spokes and inner dynein arms (IDAs) (Heuser et al., 2012; Urbanska et al., 2015). Cilia- and flagella-associated protein 61 (CFAP61), CFAP91 and CFAP251 are the mammalian orthologs of *C. reinhardtii* CSC proteins (Dymek and Smith, 2007). All these three genes showed high expressions in testis, with lower levels in the respiratory system, thyroid, female reproductive system, etc., in humans (GTEx Consortium, 2013; Fagerberg et al., 2014), suggesting a potentially important role of CSC for sperm flagella. Biallelic mutations in *CFAP91* and *CFAP251* have been reported in MMAF-affected individuals (Auguste et al., 2018; Kherraf et al., 2018; Li et al., 2019; Martinez et al., 2020). Moreover, *Cfap61* knockout mice displayed multiple morphological and ultrastructural abnormalities, severely reduced sperm count and motility, and male infertility (Huang et al., 2020). Nonetheless, whether *CFAP61* variants are also associated with MMAF in humans and the clinical manifestations of the *CFAP61* genetic anomalies have not been fully explored.

In this study, we recruited three unrelated Pakistani families, having 11 primary infertile individuals diagnosed with MMAF without manifesting any primary ciliary dyskinesia (PCD) symptoms. WES and bioinformatics analyses identified a homozygous frameshift variant (c.451_452del, p. I151Nfs*4) and a homozygous stop-gain variant (c.847C > T, p. R283*) in *CFAP61* responsible for MMAF in one and two families, respectively. Transmission electron microscopy (TEM) analyses and immunofluorescence (IF) staining revealed that these variants led to severely disorganized ultrastructures of sperm flagella with loss of CP, RSs, and IDAs in patients. Interestingly, the signals of CSC proteins CFAP61 and CFAP251 were absent from flagella of the patients. These observations suggested that these variants in *CFAP61* are pathogenic for MMAF and human male infertility.

## Materials and Methods

### Editorial Policies and Ethical Considerations

This study was approved by the Ethical Committee of University of Science and Technology of China (USTC). Written informed consent forms from all participants were obtained at the beginning of the study.

### Participants

This study recruited three Pakistani families, family 1, 2 and 3, having four (P1-P4), four (P5-P8), and three (P9-P11) infertile brothers, respectively. Semen analysis, including semen volume, sperm concentration, and sperm motility, were performed at least twice for P1-P5, P7, P9, and P10 according to the World Health Organization (WHO) guidelines (World Health Organization, 2010). Semen smears were prepared from fresh semen samples on clean glass slides, air-dried, fixed in 4% paraformaldehyde, and stored at −20°C for subsequent hematoxylin and eosin (H&E) staining or IF staining. For H&E staining, smears were washed by PBS once, stained in hematoxylin for 20 min, washed by ddH_2_O once. and put into 70% ethanol with 1% HCl for 2 s. After washing in tap water for 3 min, the slides were gradiently dehydrated by ethanol. Then the smears were stained with eosin and then placed in the baths of 100% ethanol and xylene. After sealed with neutral balsam, the smears were observed under a light microscope. Morphological analysis was performed for P1-P9 with at least 200 spermatozoa for each experiment examined (for P6 and P7, two independent experiments were performed). The semen samples for fertile control were obtained from the volunteers at the First Affiliated Hospital of USTC. A detailed questionnaire regarding PCD clinical features was obtained from all available family members and all excluded the history or presence of ciliary-related symptoms, including situs ambiguous, unexplained chronic airway infections, respiratory distress at newborn, chronic middle ear infections, clubbing, hydrocephalus etc (according to the National Institute of Health, https://www.nhlbi.nih.gov/health-topics/primary-ciliary-dyskinesia).

### WES and Variant Filtering

QIAamp Blood DNA Mini Kit (QIAGEN) was used to extract the blood genomic DNA from available family members. For WES, AIExome Enrichment Kit V1 (iGeneTech, Beijing, China)-captured libraries were constructed for family members of Family 1 (I:2, II:2, II:4, II:5, II:6 and II:7), Family 2 (I:1, I:2, II:3, II:4, II:5 and II:6), and Family 3 (III:2 and IV:1) as instructed by the manufacturer. Sequencing was carried out on a Hiseq2000 platform (Illumina, San Diego, CA, United States). Variants following recessive inheritance were kept for further screening following the strategy as we described previously (Yin et al., 2019; Zhang et al., 2020), and as shown in Supplementary Figure S1. Sequences of primers used for Sanger sequencing are listed in Supplementary Table S1.

### Electron Microscopy Analyses

Fresh semen samples were obtained and centrifuged at 900 x g for 3 min. After washing with PBS for three times, spermatozoa were fixed in 0.1 M phosphate buffer (pH 7.4) containing 4% glutaraldehyde, 4% paraformaldehyde, and 0.2% picric acid at 4°C for at least overnight. Scanning electron microscopy (SEM) and TEM analyses were conducted as we previously described (Ma et al., 2021).

### IF Staining

IF staining were performed as we previously reported (Zhang et al., 2020). Briefly, the fixed smears were taken out from −20°C for 1 h to allow to air dry at room temperature. After permeabilized with 0.2% Triton X-100 in PBS for 30 min, the smears were blocked with 3% skim milk, and then incubated with primary antibodies at 4°C overnight, followed by secondary antibodies at 37°C for 1 h. For negative control experiments, semen smears from a fertile man were stained with the mouse IgG antibodies (as the negative control for the anti-α-Tubulin antibodies) or rabbit IgG antibodies (as the negative control for the anti-SPAG6, anti-TSGA2, anti-DNAH1, anti-CFAP61, and anti-CFAP251 antibodies), followed by incubation with corresponding secondary antibodies under the same staining conditions (Supplementary Figure S2). The antibodies that were used are listed in Supplementary Table S2.

## Results

### MMAF Patients From Three Unrelated Pakistani Families

**FIGURE 1:**
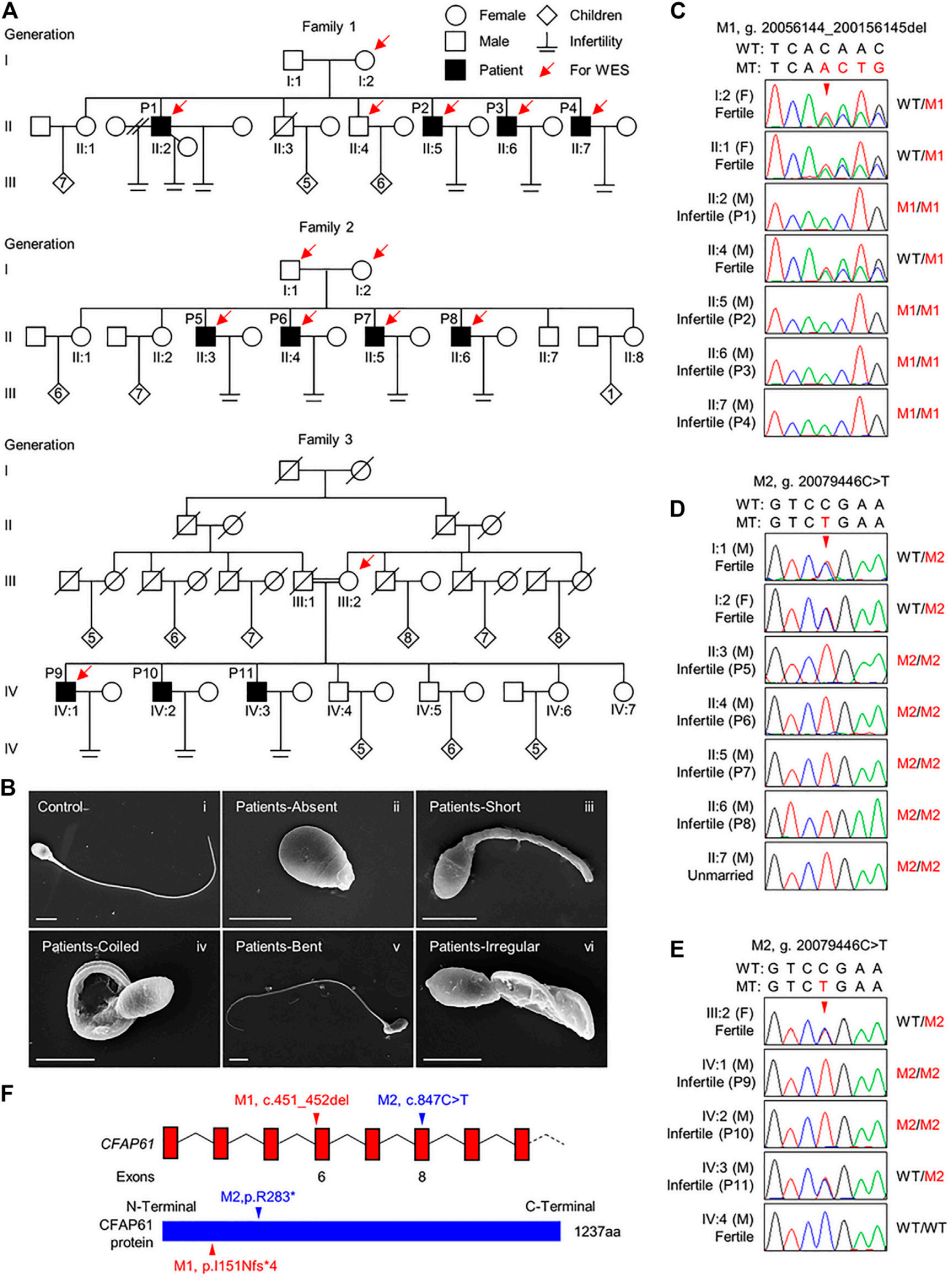
*CFAP61* variants identified in three Pakistani families with MMAF. **(A)** Pedigrees of family 1 with four infertile patients, P1 (II:2), P2 (II:5), P3 (II:6), and P4 (II:7), family 2 with four infertile patients, P5 (II:3), P6 (II:4), P7 (II:5), and P8 (II:6) and family 3 with three infertile patients, P9 (IV:1), P10 (IV:2), and P11 (IV:3). Arrows point to the individuals for which whole-exome sequencing was performed. Slashes denote deceased family members, double slash means divorce, and double horizontal lines represent consanguineous marriage. **(B)** Representative SEM micrographs showing sperm morphological abnormalities observed in patients, including absent (ii), short (iii), coiled (iv), bent (v) flagella, and flagella of irregular caliber (vi). A representative spermatozoon with normal morphology from the fertile control was shown (i). Scale bars represent 4 μm. **(C–E)** Verification of the *CFAP61* variants, p. I151Nfs*13 (NC_000020.10:g. 20056144-200156145del) in family 1 **(C)** and p. R283* (NC_000020.10:g. 20079446C > T) in family 2 **(D)** and family 3 **(E)** by Sanger sequencing using genomic DNA from all the available family members. Arrowheads, the mutation sites. F, female; M, male. WT, wild-type allele. MT, the mutant allele. M1, c.451_452del. M2, c.847C > T. **(F)** The positions of the two variants in *CFAP61* at transcript level (NM_015585.4) and protein level (NP_056400.3).

Eleven infertile men from three unrelated Pakistani families were investigated in the current study. Family 1 has four infertile brothers, P1, P2, P3, and P4; family 2 has four infertile brothers, P5, P6, P7, and; family 3 has three infertile brothers, P9, P10, and P11, born to a consanguineous marriage (Figure 1A). All the patients had been married and trying to conceive for at least 8 years, but were infertile. They had a normal karyotype (46; XY) and no large-scale deletions in Y chromosomes. According to WHO guidelines (World Health Organization, 2010), the semen volume was either normal or slightly decreased in these 11 patients. P3 had a low sperm concentration (6 million/ml) and sperm concentrations were all in the normal range for the rest of patients. However, all the patients had asthenozoospermia, with <40% sperm motility or <32% progressive motility, except for P8, whose sperm motility and progressive motility were found normal in the routine semen analysis (Table 1). Sperm morphological analysis was performed for P1-P9, and revealed that less than 3.3% of spermatozoa were morphologically normal according to WHO guidelines (Table 1). Sperm morphology was not evaluated for P10 and P11, who refused to do any more test. Hence, P1-P7 and P9 were diagnosed with asthenoteratozoospermia, P8 was diagnosed with teratozoospermia, while P10 and P11 were diagnosed with asthenozoospermia with sperm morphology undetermined.

**TABLE 1:** Clinical characteristics of patients.

	Reference values	Family 1				Family 2				Family 3		
		P1	P2	P3	P4	P5	P6	P7	P8	P9	P10	P11
cDNA mutation	—	M1/M1	M1/M1	M1/M1	M1/M1	M2/M2	M2/M2	M2/M2	M2/M2	M2/M2	M2/M2	WT/M2
Age (y)	—	51	44	41	37	39	35	31	29	40	36	38
Age (y) of marriage	—	19/25/39	30	28	27	23	20	18	21	29	28	30
Height/weight (cm/kg)	—	171.0/70.0	174.0/60.0	167.6/80.0	162.6/75.0	160.0/55.0	180.0/67.0	174.0/59.0	153.0/65.0	179.8/70.0	170.2/70.0	170.2/70.0
Semen parameters												
Semen volume (ml)	>1.5	2.0 ± 0	1.0 ± 0	2.8 ± 0.3	2.5 ± 0	2.6 ± 0.1	3.0	3.0 ± 1.5	1.8	2.6 ± 0.1	2.5 ± 0.5	2.0
Sperm concentration (10^6^/ml)	>15	73.0 ± 5.7	55.0 ± 20.0	6.0 ± 4.0	49.0 ± 29.0	27.8 ± 12.3	41.0	27.0 ± 15.0	47.0	26.3 ± 5.2	65.0 ± 17.6	20.0
Semen pH	Alkaline	Alkaline	Alkaline	Alkaline	Alkaline	Alkaline	Alkaline	Alkaline	Alkaline	Alkaline	Alkaline	Alkaline
Normal sperm morphology (%)	>4	3.3	0.6	2.3	2.6	2.3	1.2 ± 0.4	3.2 ± 1.0	1.4	2.4	ND	ND
Motile sperm (%)	>40	36.7 ± 1.7	26.0 ± 4.0	24.0 ± 4.0	15.5 ± 9.5	31.5 ± 13.5	45.0	33.5 ± 6.5	65.0	36.3 ± 6.6	41.7 ± 9.3	45.0
Progressively motile sperm (%)	>32	16.7 ± 1.7	13.5 ± 1.5	13.0 ± 5.0	3.0 ± 2.0	23.5 ± 11.5	30.0	22.5 ± 7.5	45.0	21.3 ± 3.8	25.0 ± 5.0	25.0
Sperm flagella												
Morphologically normal (%)	—	13.2	3.1	10.9	17.8	7.2	9.1 ± 5.1	10.0 ± 1.4	9.3	16.2	ND	ND
Absent (%)	—	4.8	3.5	9.8	1.6	8.5	10.7 ± 4.7	6.9 ± 5.7	1.7	4.7	ND	ND
Short (%)	—	13.2	12.9	21.9	12.4	11.5	20.0 ± 1.0	15.5 ± 0.9	16.2	16.8	ND	ND
Coiled (%)	—	21.3	45.0	21.5	16.4	28.0	10.5 ± 1.0	23.1 ± 1.9	31.4	17.0	ND	ND
Bent (%)	—	22.8	9.4	14.4	18.6	17.8	14.5 ± 0.5	19.1 ± 5.4	9.7	18.5	ND	ND
Irregular caliber (%)	—	24.7	26.1	21.5	33.2	27.0	35.2 ± 1.8	25.4 ± 0.9	31.7	26.8	ND	ND

Data with more than one experiments are presented as mean ± SEM., Reference values are shown according to WHO (the fifth edition).

Ages represents current age at submission (2021). M1, c.451_452del. M2, c.847C > T. ND, not determined.

The morphology of spermatozoa from P1-P9 was further assessed by H&E staining of semen smears. Comparing to the long and threadlike flagella observed in a fertile control, more than 82.2% of the spermatozoa in P1-P9 displayed malformed morphologies of flagella, including absent, short, coiled, bent, and irregular caliber, which are typical presentations of MMAF (Table 1 and Supplementary Figure S3). Moreover, the SEM analysis results of spermatozoa from patients were consistent with those of light microscopy (Figure 1B).

In addition, all the patients declared they have no ciliary-related clinical features (including situs ambiguous, unexplained chronic airway infections, respiratory distress at newborn, chronic middle ear infections, clubbing, hydrocephalus etc.) and none of them wish to participate in any further related examinations. Altogether, all the 11 patients exhibited male infertility due to asthenozoospermia and/or teratozoospermia associated with MMAF.

### Homozygous Variants in *CFAP61* Were Found in Patients

To explore the underlying genetic cause of MMAF in these patients, we carried out WES of P1-P4, their fertile brother II:4 and mother I:2 from family 1; P5-P8, their father I:1 and mother I:2 from family 2; P9 and his mother III:2 from family 3. Through WES data analysis, we identified a homozygous frameshift variant in *CFAP61* (M1), NM_015585.4: c.451_452del (NP_056400.3: p. I151Nfs*13), in family 1, and a homozygous *CFAP61* stop-gain variant (M2), NM_015585.4: c.847C > T (NP_056400.3: p. R283*) in family 2 (Supplementary Figure S1). In family 3, 17 variants in 17 genes, including the *CFAP61* variant same with that in family 2, were left after variant filtration (Supplementary Figure S1). Among these 17 genes (Supplementary Table S3), the *CFAP61* c.847C > T, p. R283* variant is the most likely pathogenic variant. In the gnomAD database, the allele frequencies of the *CFAP61* M1 and M2 are extremely low (0 and 0.00004017, respectively). Among the seven different ethnic groups of gnomAD, the allele frequency of M2 is 0.00023 in South Asians, 0.00016 in East Asians, and was absent in the other ethnic groups. Subsequent Sanger sequencing of all the available family members confirmed that the *CFAP61* variants were recessively inherited from their heterozygous parents except P11 whose sperm morphology was not yet assessed (Figures 1C–E). These two *CFAP61* variants, c.451_452del and c.847C > T, occurring in exon 6 and exon 8, respectively, introduce a premature stop codon and thus are expected to produce either truncated proteins lacking the vast majority of the amino acid residues or no proteins due to nonsense-mediated mRNA decay (Figure 1F). Given that *Cfap61* knockout mice was reported to have sperm morphological defects characteristics of MMAF, it is thus suggested that these two *CFAP61* variants are likely pathogenic for the MMAF phenotype observed in the patients recruited by us.

Variants in *CFAP61* led to severe disorganizations of the axonemal structures in patients.

**FIGURE 2:**
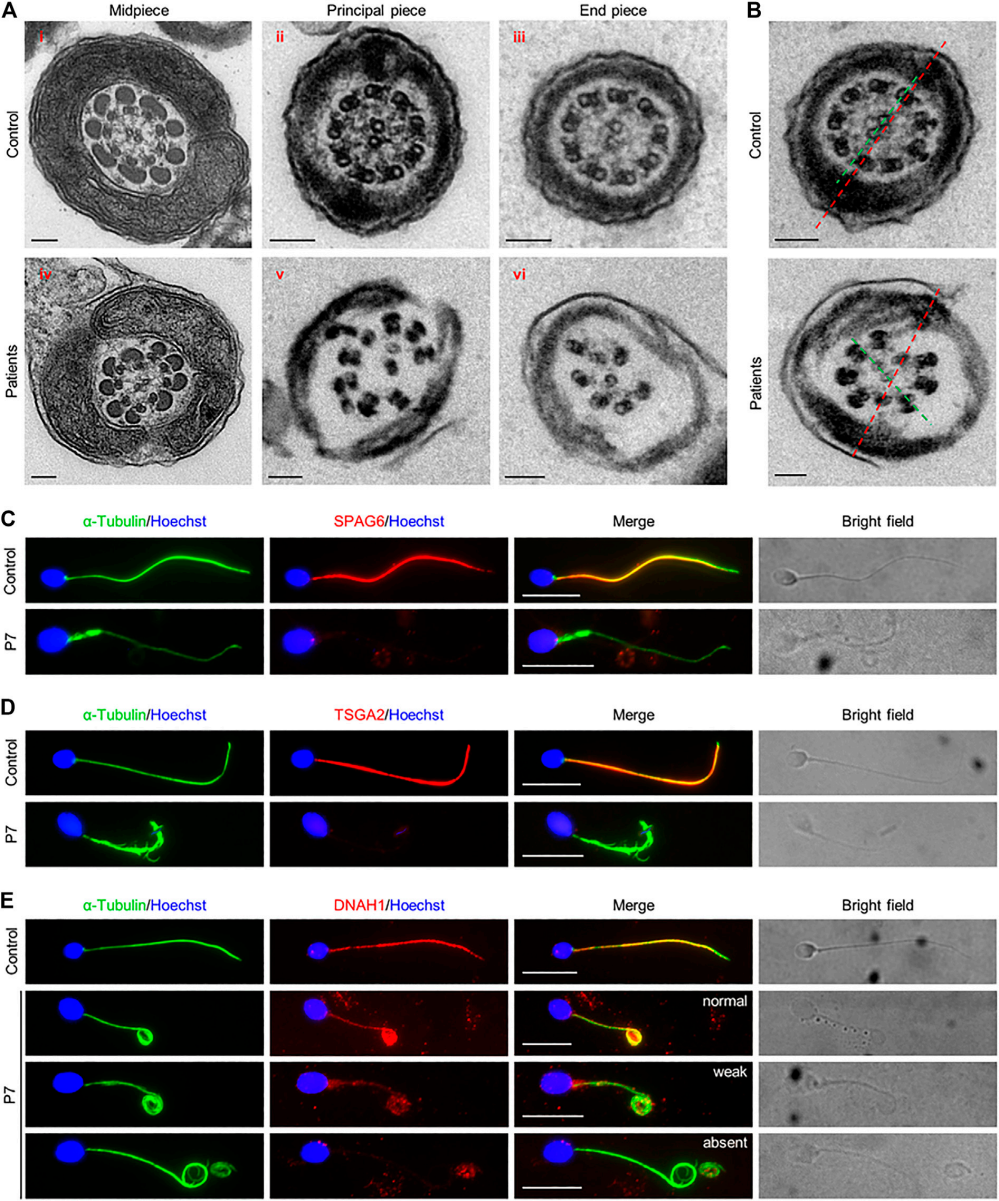
Ultrastructural flagellar abnormalities and flagellar protein defects in patients. **(A)** Representative TEM micrographs showing cross sections of midpiece (i and iv), principal piece (ii and v), and end piece (iii and vi), of sperm flagella from a fertile control and patients. Scale bars represent 100 nm. **(B)** Cross-sections showing the rotated central pairs (CPs) from patients. Red dash lines represent the expected direction of CPs, predicted from the position of longitudinal columns. Green dash lines show the observed direction of CPs, which has an angle with the expected direction (red dash line) in patients. **(C–E)** Representative images of spermatozoa from a fertile control and P7 co-stained by anti-α-Tubulin antibodies and anti-SPAG6 antibodies **(C)**, anti-TSGA2 antibodies **(D)**, or anti-DNAH1 antibodies **(E)**. Representative flagella with relatively normal, weak, or absent DNAH1 signals in spermatozoa from P7 were shown in **(E)**. Scale bars represent 10 μm.

Since disruption of *Cfap61* in mice was associated with the abnormalities of flagellum components (Huang et al., 2020), we thus investigated the ultrastructure of spermatozoa from the patients (P1-P6, P8, and P9) by TEM. In contrast to the typical “9 + 2” axoneme arrangement in the spermatozoa from a fertile control, most of the flagellar cross-sections from spermatozoa of patients exhibited severely disorganized axoneme structure, along with the missings of MTDs, dynein arms, RSs, and/or CP (Figure 2A). On average, 63.2 ± 28.4% of midpiece, 71.9 ± 17.7% of principal piece, and 69.4 ± 21.1% of end piece cross-sections were abnormal (Table 2). Interestingly, among the cross-sections with an identifiable “9 + 2” axoneme conformation in patients’ flagella, 17.4 ± 13.9% displayed misoriented CP compared to zero in controls’ (Figure 2B).

**TABLE 2:** Percentage of abnormal cross-sections in patients.

	Midpiece	Principal piece	End piece	Misoriented CP
P1	50.0% (n = 2)	50.0% (n = 10)	66.7% (n = 9)	0 (n = 9)
P2	Not observed (n = 0)	85.6% (n = 13)	91.8% (n = 12)	25.0% (n = 4)
P3	Not observed (n = 0)	85.6% (n = 13)	40.0% (n = 15)	16.7% (n = 6)
P4	33.3% (n = 3)	42.9% (n = 7)	42.9% (n = 7)	9.1% (n = 11)
P5	75.0% (n = 4)	71.9% (n = 32)	71.4% (n = 7)	7.7% (n = 13)
P6	87.5% (n = 7)	89.8% (n = 40)	90.0% (n = 28)	44.4% (n = 18)
P8	33.3% (n = 9)	65.3% (n = 72)	82.8% (n = 29)	10.0% (n = 40)
P9	100.0% (n = 9)	83.9% (n = 31)	71.4% (n = 14)	25.0% (n = 12)
Average (mean ± SD)	63.2 ± 28.4% (n = 34)	71.9 ± 17.7% (n = 218)	69.4 ± 21.1% (n = 121)	17.2 ± 13.9% (n = 113)

For analyzing the percentage of cross-sections with misoriented central pair (CP), all the cross-sections (regardless of midpiece, principal piece, or end piece) with an identifiable “9 + 2” axoneme conformation in each patient were scored. n, indicates the number of cross-sections scored.

To further investigate the ultrastructural defects observed by TEM, SPAG6, a marker of CP, TSGA2, a marker of RS, and DNAH1, a marker of IDA, were examined on the semen smears from P7. IF staining results revealed signals of SPAG6, TSGA2, and DNAH1 along the full length of sperm tails from a fertile control. In contrary to this, SPAG6 and TSGA2 signals were completely absent in the patient’s spermatozoa (Figure 2C&D). DNAH1 signals were also absent from most of the spermatozoa that were examined, and only 4.8% of spermatozoa displayed normal and 14.4% displayed weak DNAH1 signals (Figure 2E). Thus, these findings confirmed that the axoneme components, CP, RS, and IDA were severely disrupted due to the variants in *CFAP61* in patients.

Taking the above results together, patients carrying the *CFAP61* variants exhibited decreased sperm motility and morphological and ultrastructural anomalies of sperm flagella, consistent with the phenotype of *Cfap61* knockout mice (Huang et al., 2020), indicating that these two *CFAP61* variants are indeed pathogenic for MMAF in these patients.

### Variants in *CFAP61* Led to Loss of CSC Proteins in Spermatozoa

**FIGURE 3:**
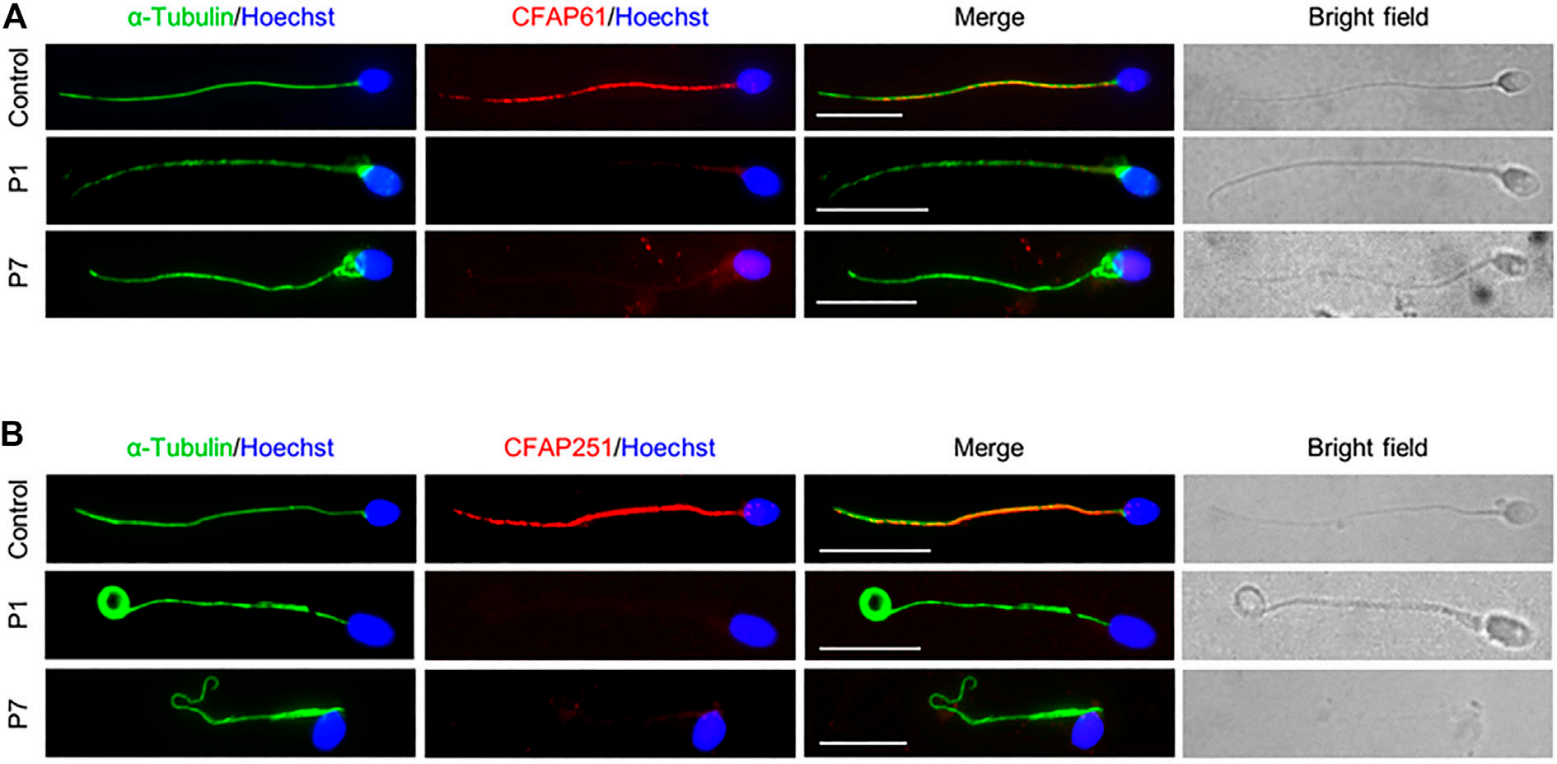
CSC proteins were not observed in sperm flagella of patients. Representative images of spermatozoa from a fertile control, P1, and P7 co-stained by anti-α-Tubulin antibodies and anti-CFAP61 antibodies **(A)** or anti-CFAP251 antibodies **(B)**. Scale bars represent 10 μm.

To confirm the pathogenicity of the identified *CFAP61* variants on protein expression, we performed IF staining on semen smears obtained from P1 and P7 with an anti-CFAP61 antibody which recognizes human CFAP61 amino acids 689–834. The specific signals of CFAP61, co-localizing with α-Tubulin, were detected on the sperm tails from a fertile control, but CFAP61 signals were absent in the spermatozoa of both patients (Figure 3A), indicating the absence of full-length CFAP61 proteins in the patients.

Since CFAP61 is a member of CSC, and spermatozoa from patients carrying variants in *CFAP61* displayed similar defects to those carrying variants in other members of CSC (Auguste et al., 2018; Kherraf et al., 2018; Li et al., 2019; Martinez et al., 2020), we next detected CFAP251 in the spermatozoa from patients. Using IF staining, we observed no CFAP251 signal in spermatozoa from patients in contrast to the signal along the full length of the flagella in spermatozoa from control (Figure 3B). These results collectively suggested that the CSC proteins are likely absent from sperm flagella of patients.

## Discussion

In this study, we identified two *CFAP61* variants from three Pakistani families with 11 infertile men suffering from MMAF, thus establishing *CFAP61* as an MMAF-related gene that is essential for functional sperm flagella. Moreover, we found that patients carrying the *CFAP61* variants displayed multiple abnormalities in ultrastructure of axoneme, including disorganization, loss of microtubules, dynein arms, and RSs, and misoriented CP. Moreover, the *CFAP61* truncating mutations led to the loss of CSC proteins in sperm flagella of patients.

In mice, *Cfap61* mRNA was detected only in testis, and *Cfap61* knockout mice displayed defects in spermatogenesis and were not mentioned of ciliary-related phenotypes (Huang et al., 2020). The patients carrying *CFAP61* variants in our study displayed almost same phenotype with the knockout mice, including no ciliary-related symptoms, reduced sperm motility, and a typical MMAF phenotype with defects in sperm morphology and axoneme ultrastructure (Huang et al., 2020). However, minor differences were found in the sperm flagella ultrastructure between patients and knockout mice. It was reported that CFAP61 was detected only at midpiece in mouse mature spermatozoa, which is consistent with the hypertrophy and hyperplasia of mitochondria in the knockout mice (Huang et al., 2020). However, in humans, CFAP61 localized along the full-length of mature spermatozoa, and we did not observe any obvious defects in the mitochondria of patients. Thus, it is likely that CFAP61 may have divergent functions in humans and mice, leading to the differences found in sperm flagella between patients and knockout mice.

Among the other three CSC protein members, variants in *CFAP251* and *CFAP91* have been reported in infertile men with MMAF (Auguste et al., 2018; Kherraf et al., 2018; Li et al., 2019; Martinez et al., 2020). Similar to CFAP61, CFAP251 also localizes throughout the whole sperm flagella in humans. Patients harboring loss-of-function *CFAP251* variants manifested severe defects in the ultrastructure of sperm flagella including disorganized ODFs, fiber sheath (FS), mitochondrial sheath (MS), and central axoneme (Kherraf et al., 2018). Though the localization pattern of CFAP91 was yet uncharacterized, MMAF patients carrying *CFAP91* variants displayed defects in ultrastructure of sperm flagella including absence of CP, and IF staining also showed partially loss of CP protein SPAG6 and RS protein RSPH1 (Martinez et al., 2020). In our study, the patients carrying *CFAP61* variants showed severe disorganizations of the axonemal structures with loss of CP, RSs, and IDAs, which was confirmed by IF staining of SPAG6, TSGA2, and DNAH1. The IF results showed a total absence of SPAG6 and TSGA2, which is more severe than the observations in patients carrying *CFAP91* variants (Martinez et al., 2020). Furthermore, we observed a complete absence of CFAP251 proteins in the sperm tails of the patients with *CFAP61* variants, indicating that CFAP61 is essential for the localization of CFAP251 in sperm flagella. Altogether, it is suggested that CFAP61 may play a more predominant role in CSC.

Interestingly, we found that about 17% of cross-sections displayed misoriented CPs, in contrast to CPs paralleling to the axis of the two longitudinal columns of the FS observed in controls. It was reported that RS is important for the anchorage of the CP, and mutation in RS proteins, like RSPH4A or RSPH9, will cause the rotation of CP, which might lead to the degeneration of CP (Burgoyne et al., 2014). In the present study, we found that RSs were absent in patients’ flagella. Moreover, the rotation of CPs and loss of CPs were also found after disruption of CFAP91, another component protein of the CSC, in *T. brucei* cells (Martinez et al., 2020). Thus, we supposed that CSC may be required to ensure the correct orientation and stability of CP in an indirect way.

Altogether, our data demonstrate that CFAP61 is essential for normal sperm flagellum structure and function and variants in *CFAP61* affect the normal assembly of CP, RSs, and IDAs, leading to MMAF in humans. These findings will expand the current understanding in the disease-causing mutations and the associated flagellar phenotypes of MMAF and provide guidance for genetic counseling and diagnosis of the disease.

## Data Availability

The WES data presented in the study are deposited in the Genome Variation Map in BIG Data Center, Beijing Institute of Genomics (BIG), Chinese Academy of Sciences, accession number GVM000292. All other data are included in the article/Supplementary Material.
