# Supplementary figures and images for: Biallelic Variants in CFAP61 Cause Multiple Morphological Abnormalities of the Flagella and Male Infertility
Source: Front Cell Dev Biol. 2022 Jan 31;9:803818. doi: 10.3389/fcell.2021.803818 (PMC8841411; doi:10.3389/fcell.2021.803818)

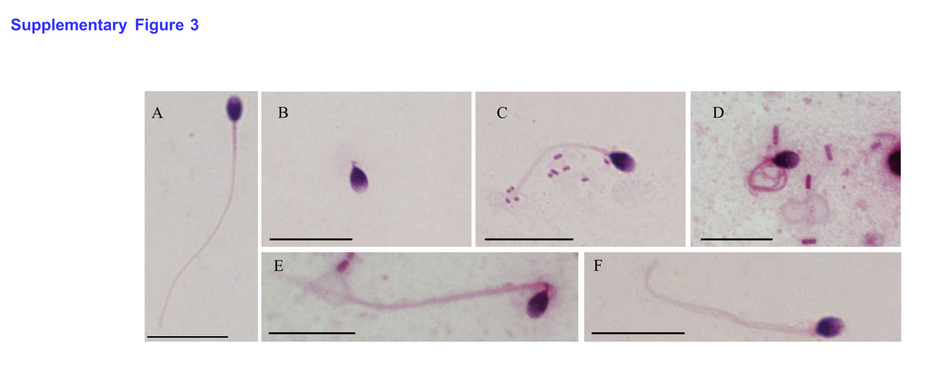

Supplement: Supplementary file 2 [file Image3.TIF]

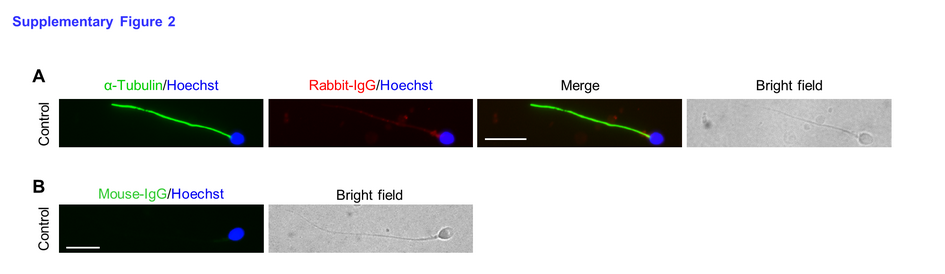

Supplement: Supplementary file 3 [file Image2.TIF]

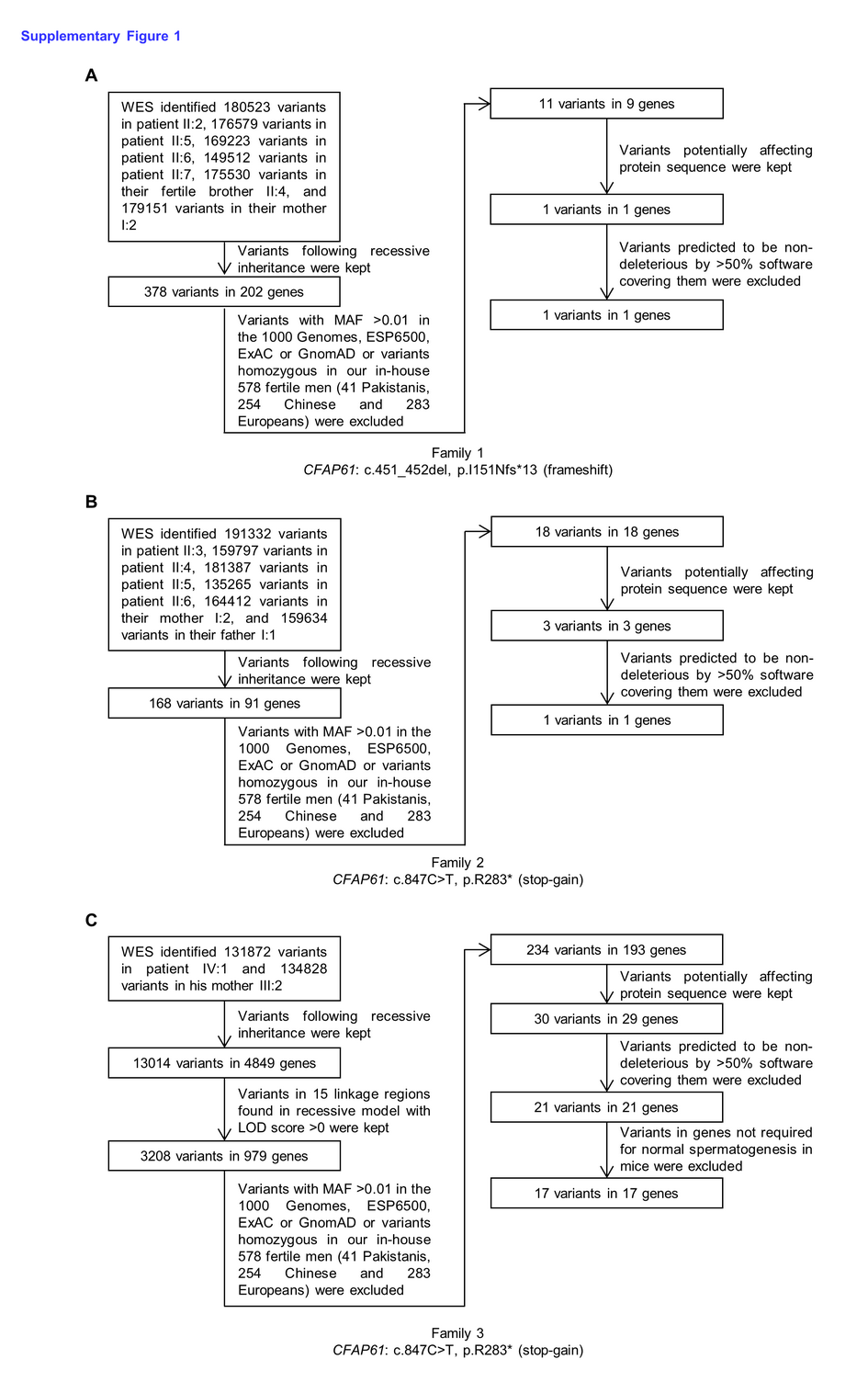

Supplement: Supplementary file 4 [file Image1.TIF]
